# Prediction of the Transprosthetic Velocity Following Aortic Valve Replacement with Bioprosthesis in the Patients with Aortic Stenosis

**DOI:** 10.1186/1749-8090-10-S1-A246

**Published:** 2015-12-16

**Authors:** Yukio Umeda, Yasunari Yokota, Yoshio Mori, Yoko Kawamura, Hiroshi Takiya

**Affiliations:** 1 Department of Cardiovascular Surgery, Gifu Prefectural General Medical Center, Gifu City, Gifu, 500-8717, Japan; 2 Faculty of Engineering, Gifu University, Gifu City, Gifu, 501-1193, Japan; 3 Research and Development Center for Human Medical Engineering, Gifu University, Gifu City, Gifu, 501-1193, Japan

## Background/Introduction

In aortic valve replacement(AVR), to obtain the largest possible effective orifice area index (EOAI) is considered to be desirable to avoid prosthesis patient mismatch in patients with aortic stenosis (AS). In spite of the adequate EOAI, sustained high transprosthetic velocity was often observed in early after AVR with bioprosthesis.

## Aims/Objectives

We hypothesized that we could predict the transprosthetic velocity following AVR by clarifying the effect of AVR on cardiac function and peripheral vascular distensibility.

## Method

Twenty patients with AS without aortic insufficiency underwent AVR using bioprostheses were recruited. Pre- and post-AVR cardiac function parameters and aortic valve parameters were collected by transthoracic echocardiography. To evaluate the influences of peripheral vascular resistance on transvalvular flow velocity, static distensibility parameters of the carotid artery, such as β stiffness index, pressure-strain elastic modulus (Ep), and arterial compliance (AC), were obtained by a real time echo-tracking system at pre-AVR and 1 week after AVR.

## Results

Calculated effective orifice area index (EOAI) of the prosthetic valve was 1.13 ± 0.18. Pre-AVR peak AoV and post-AVR peak transprosthetic velocity was 5.15 ± 0.82 m/s and 2.78 ± 0.59 m/s, respectively. There was significant reduction in peak AoV following AVR. With regards to peripheral vascular resistance, static distensibility parameters of the carotid artery were not affected by AVR (pre- vs post-AVR; β stiffness index, 15.26 ± 6.61 vs 15.07 ± 7.74, Ep, 190.46 ± 83.70 kPa vs 194.69 ± 104.15 kPa, AC, 0.66 ± 0.32 mm2/kPa vs 0.67 ± 0.31 mm2/kPa). No significant changes in LVEF and FS were also found. There was significant correlation between the ratio of peak transprosthetic velocity to pre-AVR peak AoV and the ratio of calculated EOAI to pre-AVR AVAI. As a result, predicted transprosthetic peak velocity could be calculated as (0.7642-0.084 × EOAI /pre-AVR AVAI) × pre-AVR peak AoV.

## Discussion/Conclusion

We explored the effects of AVR on cardiac function and peripheral vascular distensibility. And we found a formula to predict the transprosthetic velocity following AVR.

